# 1927. Use of a SARS-CoV-2 Strand-specific Assay to evaluate for Prolonged Viral Replication among Hospitalized Patients. Tertiary Care Center, California 2020–2022

**DOI:** 10.1093/ofid/ofac492.1554

**Published:** 2022-12-15

**Authors:** Jessica Ferguson, Gustavo A Contreras Anez, Lucy S Tompkins, John Shepard, Ayelet Rosenthal, Aruna Subramanian, Benjamin A Pinsky, Jorge Salinas

**Affiliations:** Stanford University, Palo alto, California; Stanford University, Palo alto, California; Stanford University, Palo alto, California; Stanford University, Palo alto, California; Stanford University, Palo alto, California; Stanford University, Palo alto, California; Stanford University, Palo alto, California; Stanford University, Palo alto, California

## Abstract

**Background:**

Determining if a patient with SARS-CoV-2 remains infectious is an infection control challenge in healthcare settings; specially, among critically ill or profoundly immunosuppressed patients. We use an assay that detects minus-strand RNA as a surrogate for actively replicating SARS-CoV-2. We report positive strand-specific assays in relationship to time since admission and describe patients with a detectable strand-specific assay >20 days since admission.

**Methods:**

We use a 2-step rRT-PCR specific to the minus strand of the SARS-CoV-2 envelope gene. The strand-specific assay is used to evaluate for infectivity in asymptomatic patients with a positive admission screening or pre-procedural test or if ongoing replication is suspected (critical illness or profound immunosuppression). We retrieved strand-specific test results for patients hospitalized at Stanford Healthcare during August 2020–March 2022. We describe clinical characteristics for patients with a detectable minus strand-specific test >20 days since admission.

**Results:**

A total of 774 strand-specific tests were collected from 624 hospitalized patients. A total of 523 patients had only one test (84%) and 101 (16%) had ≥2 tests. The test positivity rate varied by time since admission: 19% in tests performed 0–5 days, 28% in 6–10 days, 22% in 11–20 days, and 41% in those >20 days since admission. Among 35 patients tested >20 days since admission, 13 (37%) had ≥1 detectable minus strand-specific test. Most were male (n=8, 62%) and mean age was 59. Of 13 patients with a detectable assay, seven (54%) had prolonged viral replication with persistent symptoms and detectable minus strand assays for >20 days from symptom onset. Of these seven patients, four had a transplant (3 lung, 1 liver), 1 ovarian cancer, 1 CAR-T cell therapy, and 1 ESRD without immunosuppression. The remaining eight patients with a detectable assay >20 days since admission had illness onset while hospitalized.

**Conclusion:**

Among hospitalized patients with SARS-CoV-2 infection, we found a varying positivity rate according to the timing of testing, possibly reflecting different indications for the test. The strand-specific assay may help assess for infectiousness in profoundly immunocompromised patients.

**Disclosures:**

**Aruna Subramanian, MD**, Gilead Sciences: Grant/Research Support|Regeneron, Inc: Grant/Research Support.

